# Realization of Bidirectional, Bandwidth-Enhanced Metamaterial Absorber for Microwave Applications

**DOI:** 10.1038/s41598-019-46464-6

**Published:** 2019-07-11

**Authors:** Lincy Stephen, N. Yogesh, V. Subramanian

**Affiliations:** 1 Microwave Laboratory, Department of Physics, Indian Institute of Technology Madras, Chennai, 600036 India; 2 Department of Nuclear Physics, School of Physical Sciences, University of Madras, Chennai, 600025 India

**Keywords:** Microwave photonics, Metamaterials

## Abstract

The ever-increasing interest towards metamaterial absorbers owes to its remarkable features such as ultra-thin nature and design flexibility. Subduing the inherent narrow bandwidth of such absorbers is the prime goal in metamaterial absorber research, as this can widen the applications areas. A greater challenge is to construct bidirectional absorber, which provides direction-insensitive absorption, as most of the existing designs exhibit single sided absorption due to the complete metal film used in the design. This work presents the realization of a bidirectional, bandwidth-enhanced metamaterial absorber with basic elements such as strips and squares optimized to have adjacent resonances leading to a bandwidth-enhanced absorption. The structural evolution of the constituent metallic components towards the formation of bandwidth-enhanced absorption is described. The bidirectional absorber exhibits more than 90% absorption between 13.40 GHz and 14.25 GHz from the two incident directions. The mechanism of absorption is studied with the surface current analysis and the effective parameters of the structure. The choice of the metallic components with four-fold rotation symmetry renders the proposed design to be polarization independent and wide-angle receptive. The numerical studies are verified experimentally at microwave frequencies, which shows a good agreement between them.

## Introduction

Absorption of electromagnetic (EM) radiation is indispensable in many areas such as stealth, communication and antenna. For instance, absorbers are employed for radar cross section reduction in defence applications^1^. It can be efficiently utilized for the reduction of antenna side lobes^2^ as well as the backscatter from microstrip radiators, which is integral in the design of both mobile communications and high precision systems^3^. Electromagnetic interference (EMI) shielding is another thrust area, where absorbers are widely used for the elimination of undesirable EM radiations from the electronic appliances, and this can improve the signal to noise ratio and fidelity of electronic components including switches, oscillator, amplifiers and modulators mounted on the same platform^4^. Different types of absorbers have been utilized, such as Salisbury screen^5^ and Jaumann absorber^1^ to meet the above applications. Most of these conventional absorbers consist of a resistive sheet and a metal backing, separated by a dielectric of quarter wavelength thickness to produce near unity absorption. However, there are a few limitations for these types of absorbers. One of the major difficulties is to find materials that naturally impedance-match with free space to reduce reflection. With an impedance matched absorption surface, the problem of eliminating stray EM signals in electronic circuits can be handled effectively. Hence designing an impedance matched high-absorption surface is of high importance for EMI shielding and EM compatible structures. Another limitation of conventional absorber is its weight. Since the requirement for quarter wavelength thickness of these absorbers makes EM components bulky, adding more layers for broadband absorption cannot serve for applications requiring electrically thin components.

The emergence of metamaterials (MTMs) has transformed the field of EM wave absorption in the most remarkable way. The artificial composites consisting of an array of sub-wavelength unit cells facilitate ultra-thin compact absorbers. With the user-defined ‘meta atom’ as a unit cell, MTMs offer to tailor the effective parameters such as complex permittivity and permeability by suitably varying the geometrical shape and dimensions of the unit cells leading to much more design flexibility than the conventional counterparts. Additionally, the unit cells can be selected to be polarization-insensitive and wide-angle receptive. The first demonstration of metamaterial absorber prototype by Landy *et* al. in 2008^6^ prompted an ever-increasing interest in the field leading to many metamaterial based absorbers from microwave to visible region^7–16^ for potential applications in bolometer^17^, thermal imaging^18^, energy harvesting^19,20^, sensor devices^21^, camouflaging in stealth technology and communications^22^. Amidst the compelling advantages, MTM based absorbers face some limitations. The major concern is the narrow bandwidth of operation, which is connected with the resonant nature of the design elements. This can be a hindrance in practical applications and hence requires multi-band or broadband absorbers to overcome this limitation^23–31^. A less explored and even more challenging area in metamaterial absorbers is the direction-insensitive absorption. Design of most of the metamaterial absorbers employs a metal-dielectric-metal configuration, with a complete metal film as the back layer along with the metallic patterns on the other side of the dielectric substrate^32–36^. This not only makes the design process much easier but also suppresses the transmission. However, the trade-off is that this reduces the incident direction to one, as the radiation incident from the other direction is completely reflected like from a perfect reflector.

In this work, a bidirectional bandwidth-enhanced absorber is realized by blending the resonances of the different metallic elements constituting the unit cell which is extremely challenging as the metallic components causing one resonance can hinder another one, thus complicating the selection of appropriate metallic patterns. The optimized bidirectional absorber exhibits absorption greater than 90% from 13.38 GHz to 14.4 GHz along one direction, and in the reverse direction, it exhibits near perfect absorption between 13.40 GHz and 14.25 GHz. The structural evolution responsible for the bandwidth-enhanced absorption is described to elaborate the contribution of various metallic components towards the absorption. The mechanism of absorption is explored with effective parameters and surface current analysis. Further, the polarization-insensitivity and the wide-angular response of the proposed absorbers are investigated. Finally, the simulation results are verified experimentally.

## Results

**Figure 1:**
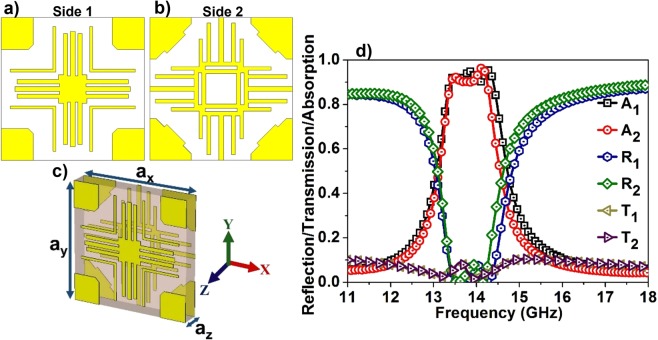
The unit cell of the bidirectional bandwidth-enhanced absorber. (**a**) Front layer (Side 1) (**b**) back layer (Side 2) (**c**) perspective view of the absorber (**d**) simulated absorption of the proposed absorber.

Figure 1(a–c) illustrates the optimized design of the proposed bidirectional bandwidth-enhanced absorber. The metallic patterns are made of copper with a thickness of 0.034 mm and conductivity of 5.8 × 10^7^ S/m. The dielectric layer is chosen as FR-4, which is a glass epoxy substrate with a thickness of 1.5 mm, dielectric constant of 4.3 and loss tangent of 0.025. The dimensions of the front and back metal patterns are tuned to maximize the absorption magnitude as well as the bandwidth, which is elaborated in the following design evolution section. The lattice constants of the unit cells are a_x_ = 10.3 mm, a_y_ = 10.3 mm and a_z_ = 1.5 mm (~λ/14 where λ is the wavelength of operation which is much less than the λ/4 thickness of the conventional absorbers). The optimized design parameters are presented in the supplementary material (See Fig. S1).

The design optimization and comprehensive numerical analysis of the absorber are carried out with CST Microwave Studio^®^. The radiation is incident along the Z-direction, and appropriate boundary conditions are applied in the perpendicular directions. The obtained scattering parameters are used for calculating the reflection and transmission as, $R(\omega )={|{S}_{11}|}^{2},\,T(\omega )={|{S}_{21}|}^{2}$ and the absorption is computed as $A(\omega )=1-{|{S}_{11}|}^{2}-{|{S}_{21}|}^{2}$. For negative Z-direction (i.e. Side 1) EM incidence, the transmission, reflection and absorption are represented as T_1_, R_1_ and A_1_ respectively whereas the corresponding terms for positive Z (i.e. Side 2) incidence is represented as T_2_, R_2_ and A_2_. Figure 1(d) shows the simulated absorption of the proposed absorber. From both the incident directions, transmission remains the same with minimal magnitude. The reflection R_1_ exhibits resonances at 13.674 GHz and 14.283 GHz, and similarly, R_2_ encompasses two resonances at 13.562 GHz and 14.143 GHz. The presence of these adjacent resonances along with the small magnitude of transmission results in a bidirectional bandwidth-enhanced absorption from the structure. Calculated absorption, A_1_, is greater than 90% from 13.38 GHz to 14.4 GHz (bandwidth of 1.02 GHz) and A_2_ also remains greater than 90% from 13. 40 GHz to 14.25 GHz (bandwidth of 0.85 GHz). The fractional bandwidth for the design is calculated as, FBW [%] = 100 × (*f*_H_–*f*_L_)/*f*_C_, where *f*_H_, *f*_L_ and *f*_C_ are highest, lowest and center frequency of the 90% absorption bandwidth respectively. The calculated FBW for side 1 is 7.3%, and for side 2 it is 6.1%.

### The design evolution of the proposed bidirectional bandwidth-enhanced absorber

To realize a bidirectional absorber, it is necessary to have metal patterns on both sides of the dielectric substrate. However, it is not achievable by mere duplication of metallic patterns. This demands utmost care in the realization of metallic patterns on the substrate, which can aid in the absorption from the two incident directions by simultaneously matching impedance as well as minimizing transmission from both the directions. The design of bidirectional broadband absorber with multiple resonating elements is even more challenging as the elements which results in one perfect absorption band can affect the other absorption bands unfavourably. Hence, the problem is approached as a two-step process, wherein a unidirectional bandwidth-enhanced absorber is designed initially, which is then modified to realize the bidirectional bandwidth-enhanced absorber.

**Figure 2:**
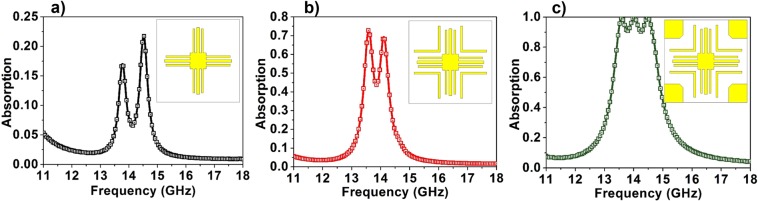
The design evolution of the unidirectional bandwidth-enhanced absorber.

For the design of the unidirectional bandwidth-enhanced absorber, metallic patterns consisting of simple elements such as metallic strips and squares are chosen on one side of the dielectric substrate and a complete metallic film on the other side of the substrate and the optimization of which leads to the bandwidth-enhanced absorption. Instead of using entirely independent resonant elements, the coupling between the elements is utilized for obtaining bandwidth enhanced absorption. Figure 2 shows the design evolution of the proposed unidirectional bandwidth-enhanced absorber. The initial step (Fig. 2(a)) shows the first element of the design which is the square in the centre (front midsq) and two strips (front str1, the strip which is placed in the middle and front str2, a wider strip in the gap of which str1 is placed) attached to the square are arranged in both horizontal and vertical directions. As can be seen from the figure (Fig. 2(a), the absorption efficiency of this element is very less (~20%) ascribed to the impedance mismatch, inducing large reflection from the structure. Despite the less absorption efficiency, the spectrum exhibits two nearby resonances (13.77 GHz and14.51 GHz) that can bring about bandwidth-enhanced absorption. Following this, another strip (front str3) wider than str2 with an optimized gap where the first element is placed, is introduced in the design. The addition of front str3 increases the absorption level (~70%) with a change in the resonant frequencies to 13.59 GHz and 14.108 GHz (Fig. 2(b)). In the final step, optimized square elements (front endsq) are incorporated at the corners of the unit cell (Fig. 2(c)). This not only improves the absorption magnitude but also widens the bandwidth. The presence of square resonators enhances the resonances induced by the strip components and produces a bandwidth-enhanced, highly efficient absorption.

**Figure 3:**
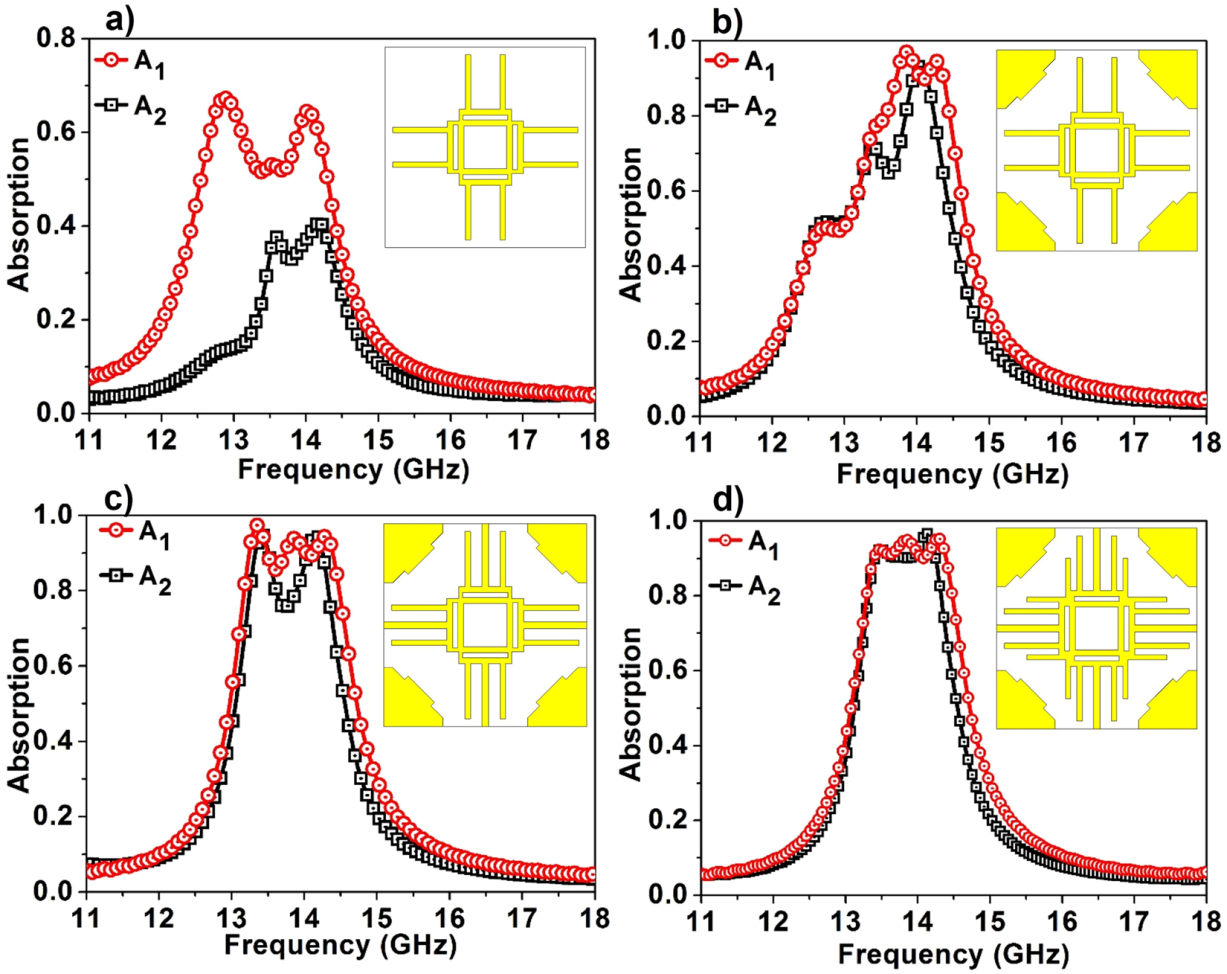
The design evolution of the proposed bidirectional bandwidth-enhanced absorber.

In the second phase of the design process, the bidirectional absorber is obtained from the unidirectional absorber by replacing the complete metallic film at one side with metallic patterns that support the existing resonances. The design procedure leading to bidirectional bandwidth-enhanced absorption is shown in Fig. 3. In the first step, a hollow square (back midsq) and a strip with a gap (back str2) are used at the back side of the dielectric substrate (Fig. 3(a)). The gapped metallic strips are placed in horizontal and vertical directions to maintain the rotational symmetry. This results in absorption A_1_ with two maxima at 12.869 GHz (67%) and 14.044 GHz (64%) whereas A_2_ contains two peaks at 13.583 GHz (38%) and 14.177 GHz (40%). Following this, squares (back endsq) with dimensions optimized are added at the four corners which improve the impedance matching in both the directions, albeit a slight increase in the transmission level. This reflects in the absorption spectrum with a higher magnitude level; A_1_ has three prominent maxima at 12.75 GHz (50%), 13.856 GHz (96.9%) and 14.276 GHz (94.5%) whereas, A_2_ has maxima at 12.743 GHz (51.8%), 13.401 GHz (71.8%) and 14.045 GHz (93%) (Fig. 3(b)). Hence, in order to decrease the transmission level while maintaining the low reflection levels, a new strip (back str1) is introduced in the design which, along with its perpendicular counterpart is placed in the gap of back str2. The new absorption spectrum (Fig. 3(c)) exhibits much more robustness in terms of absorption magnitude as well as bandwidth from both the incident directions. Absorption A_1_ displays three peaks at 13.352 GHz (97%), 13.863 GHz (93.8%), 14.29 GHz (94.46%) while maintaining absorption magnitude ≥85% for the resultant band whereas A_2_ reduces to two sharp maxima at 13.415 GHz (95%) and 14.164 GHz (94.66%). Although the peak absorption values are high, due to the sharp resonances, the intermediate region of the absorption spectrum A_2_ shows magnitude approximately equal to 75%. To further improve the A_2_ spectrum, a new strip (back str3) which has a width equal to that of the back midsq is added to the design. The dimensions and gap of the new strip, where back str1 and back str2 are placed, are optimized which leads to a near perfect absorption from both the incident directions (Fig. 3(d)).

### Numerical analysis of the bidirectional bandwidth-enhanced absorber

**Figure 4:**
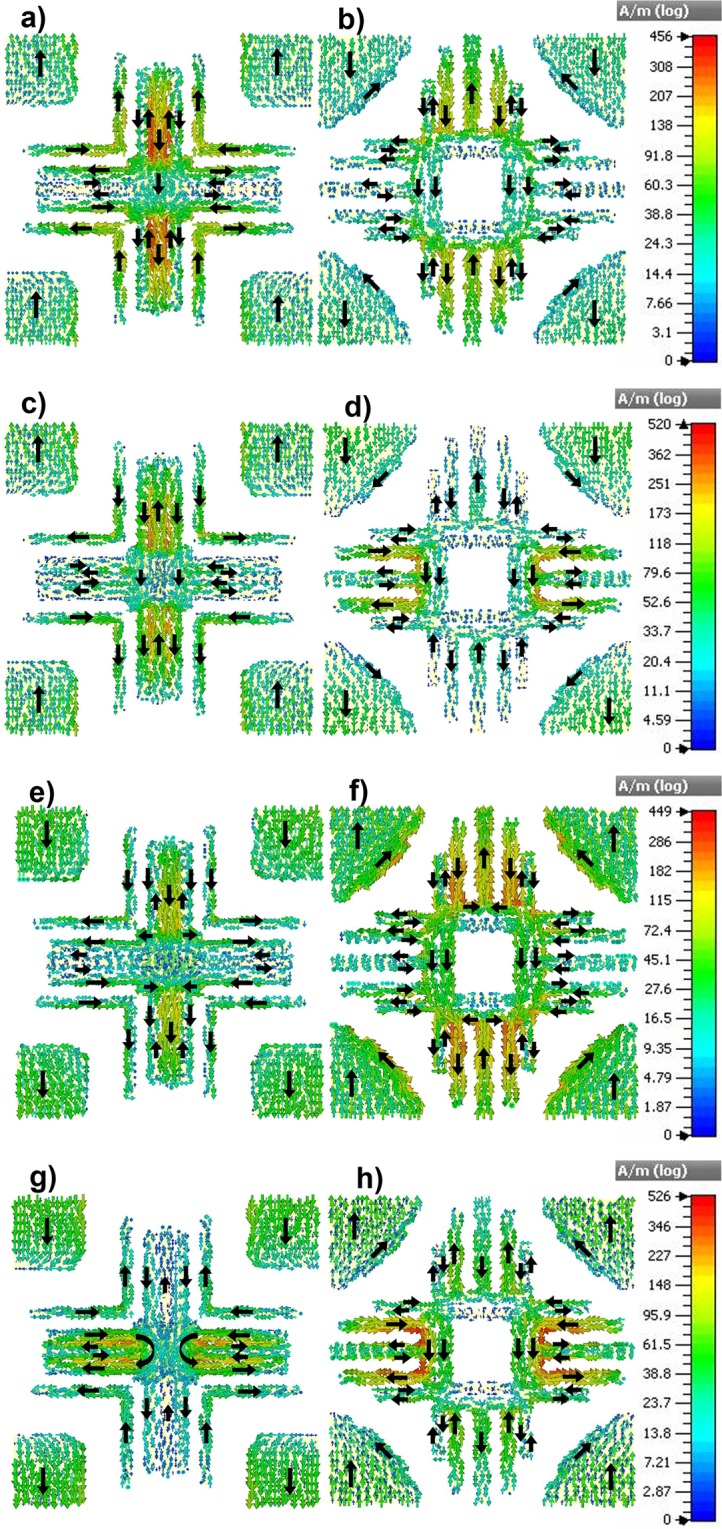
Surface current distribution on the front and back surface of the bidirectional bandwidth-enhanced absorber. At 13.674 GHz (**a,b**)), 14.283 GHz (**c,d**)), 13.562 GHz (**e,f**)) and 14.143 GHz (**g,h**)).

To explain the mechanism of absorption, surface current analysis is carried out at four different frequencies, 13.674 GHz, 14.283 GHz and 13.562 GHz, 14.143 GHz which are the resonance frequencies responsible for bandwidth-enhanced absorption from side 1 and side 2 respectively (Fig. 4). The magnitude of the current generated at all these frequencies is nearly equal with a slightly varying absorption mechanism. At 13.674 GHz (Fig. 4(a,b)), the current is mainly accumulated in the vertical arms of the strips in the front and back layers. The four squares at the corners (endsq) in the front and back layers form one segment of current flow followed by the front and back str3. The front layer elements, front str2, the vertical arm of front str1 along with the corresponding elements in the back layer including back str1 and str2 form another segment of current flow in the top and bottom halves separately. At all these segments, current flow in the front layer is anti-parallel to that of the back layer leading to a strong induced magnetic dipole response from the structure at this frequency. At the next resonance frequency 14.283 GHz (Fig. 4(c,d)), the current is mainly concentrated in the vertical arms of front str1 and str2 as well as the horizontal arms of the back str1 and back str2. Similar to the previous case, the sections constituting the four endsq on the front and back layers as well as the section including front str3 and back str3 form anti-parallel set of current in the structure resulting in the magnetic response. On the contrary, the segment which includes the front str1 and front str2 makes a parallel current with the back str1 and back str2 leading to electric dipole resonance from the structure.

At 13.562 GHz ((Fig. 4(e,f)), the first resonance frequency responsible for the absorption from side 2, the main contribution to the absorption mechanism comes from the vertical arms of the metallic elements, front and back str1 and str2. The end squares at the front and back layers as well as the vertical sections of the str1 and str2 on front and back layers form currents in opposite directions inducing magnetic response whereas horizontal arms of str1 and str2 on the front and back layer and front and back str3 form currents that are parallel to each other resulting in electric response at this frequency. At 14.143 GHz (Fig. 4(g,h)), the main contribution comes from the horizontal arms of the front and back str1 and str2, the currents generated in which are parallel in direction leading to a strong electric response in the structure. Similarly, the front and back str3 also form currents which are parallel to each other. Magnetic response is caused by the antiparallel currents in the segments of front and back endsq as well as the vertical arms of front and back str1 and str2.

**Figure 5:**
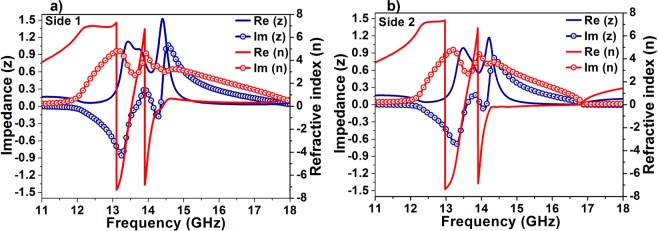
Effective impedance and refractive index of the proposed absorber from (**a**) side1 and (**b**) side 2.

To further comprehend the origin of the bidirectional bandwidth-enhanced absorption, the effective parameters of the structure are retrieved using the standard retrieval method^37^. The two conditions for perfect absorption, minimum reflection and minimum transmission can be verified by the effective parameters. The minimum reflection implies impedance matching with the surroundings, which is shown by value ~1(i.e. 377 ohms as that of air) in the normalized impedance diagram whereas, the minimum transmission suggests a large loss in the structure which is indicated by a positive value of the imaginary part of the refractive index in the effective refractive index spectrum. Figure 5(a,b) shows the effective impedance and refractive index when the radiation is incident from side 1 and side 2, respectively. Owing to the small difference in the scattering parameters from the two incident directions, the retrieved effective parameters differ slightly. The structure exhibits perfect absorption A_1_ from 13.38 GHz to 14.4 GHz for which the impedance values are in between the range 0.6 and 1.4, and the imaginary part of the refractive index is positive in the entire range with values between 2.71 and 4.61. For radiation incident from side 2, A_2_ shows near perfect absorption from 13. 40 GHz to 14.25 GHz for which the normalized effective index falls between 1.18 and 0.56 whereas the imaginary part of refractive index values is between 4.41 and 2.7. Another important point to be noted from the figure is that the dissipation factor (imaginary part of the refractive index) of the structure is much higher compared with that of the loss tangent of the lossy substrate FR-4 which is 0.025.

**Figure 6:**
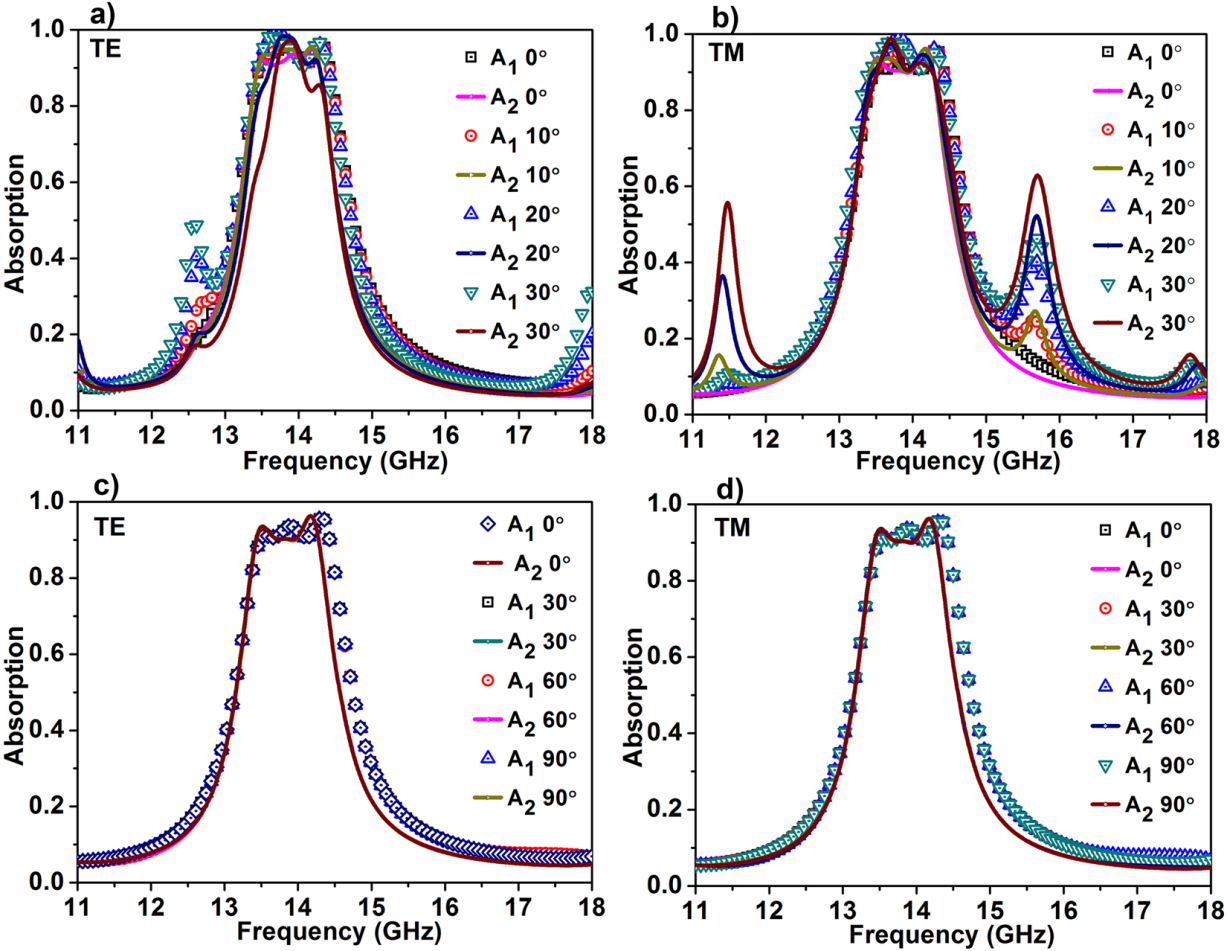
Polarization-insensitivity and wide-angle receptivity analysis of the proposed absorber. Absorption at different incident angles for (**a)** TE polarization (**b)** TM polarization and at different polarization angles for (**c)** TE polarization (**d)** TM polarization.

Further, the robustness of the absorption for different polarizations and various incident angles are analysed (Fig. 6). Owing to the four-fold symmetry of the constituting metallic patterns, the proposed absorber maintains bidirectional bandwidth-enhanced absorption for both transverse electric (TE) and transverse magnetic (TM) polarizations. For TE and TM normal incidence, A_1_ exhibits near perfect absorption greater than 90% from 13.48 GHz to 14.43 GHz (bandwidth of 0.95 GHz), and for A_2_, the absorption magnitude is greater than 90% from 13.42 GHz to 14.28 GHz (bandwidth of 0.86 GHz). As the incident angle increases, the structure retains near perfect bandwidth-enhanced absorption to some extent. For TE polarization (Fig. 6(a)), the absorption A_1_ retains strong bandwidth-enhanced absorption as the incident angle increases, while absorption A_2_ deteriorate with increasing incident angles. Up to incident angle 30° the structure shows bidirectional bandwidth-enhanced absorption with magnitude greater than 80%, after which A_2_ loses its bandwidth-enhanced nature. Comparing with TE polarization, TM polarization (Fig. 6(b)), exhibits wide angle receptivity with more than 80% up to 50°. As the angle increases, A_1_ retains its bandwidth-enhanced nature but the magnitude decreases, and for large angle of incidence, the absorption band starts to split, forming separate absorption bands. In the case of A_2_ also, the bandwidth-enhanced nature is preserved with the high magnitude, and for a large angle of incidence, additional peaks are generated. The condition for impedance matching is different for oblique incidence, and this may lead to additional peaks in the spectrum, as shown in the figure. The surface current analysis demonstrating the absorption mechanism at oblique angles is presented in the supplementary material (See Fig. S2). Overall, the proposed bidirectional absorber maintains bandwidth-enhanced perfect absorption up to 30° from both the incident directions at different polarizations.

The absorption is studied under different polarization angles for TE and TM polarizations (Fig. 6(c,d)). For both the polarizations, absorption remains the same for all the polarization angles from 0° to 90° which can be attributed to the four-fold symmetry of the proposed absorber.

## Experimental Results

**Figure 7:**
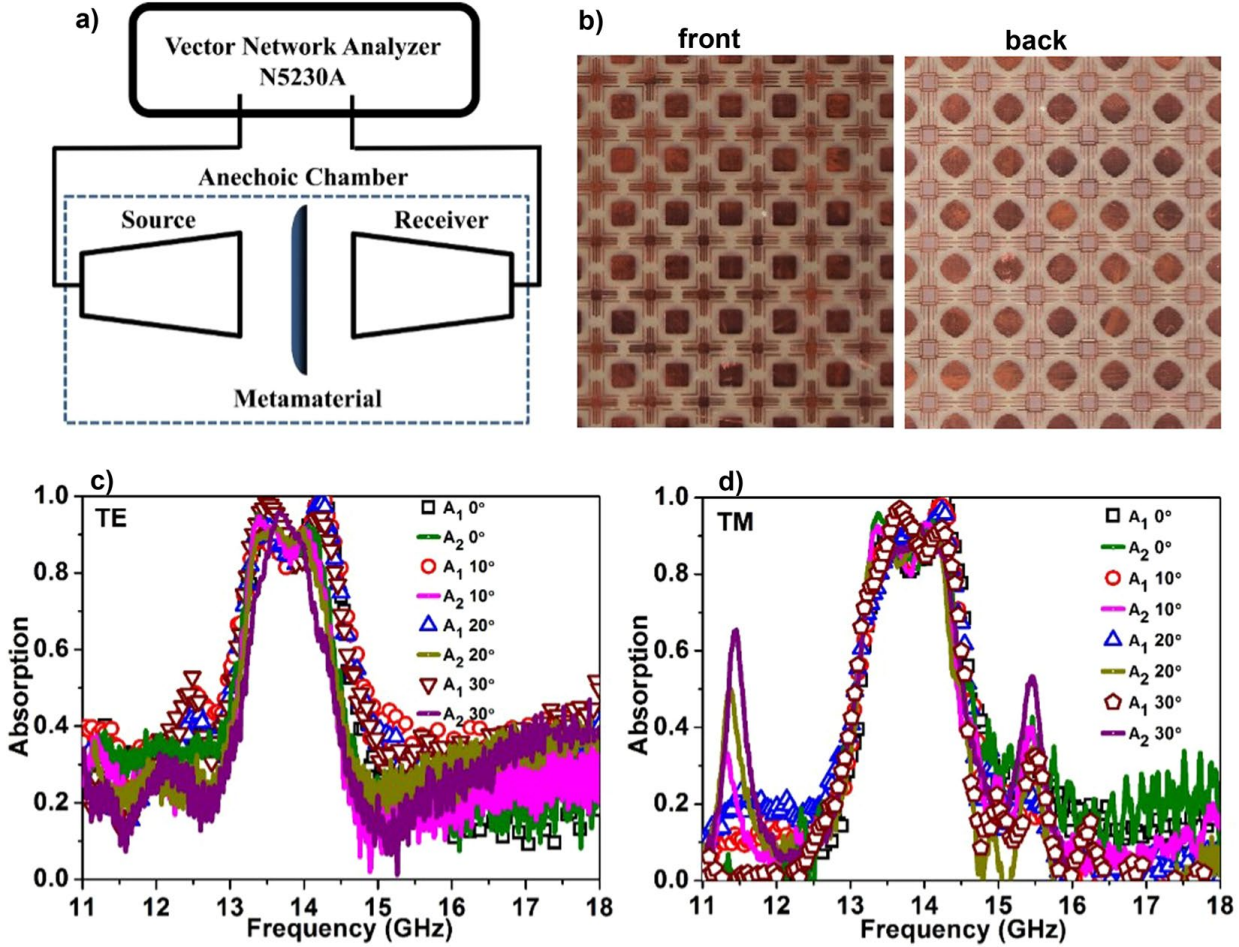
(**a**) Experimental configuration of the proposed bidirectional bandwidth-enhanced absorber. (**b**) Front and back layer of the fabricated prototype. Measured absorption at different incident angles for (**c**) TE polarization and (**d**) TM polarization.

The optimized design of the bidirectional bandwidth-enhanced absorber is fabricated on FR-4 board with 267.8 mm × 267.8 mm × 1.5 mm dimensions (Fig. 7(b)). Microwave experiment is carried out on the fabricated sample using free space method with Ku-band (12 GHz to 18 GHz) horn antennas and a network analyzer (N5230A) (Fig. 7(a)). The fabricated metamaterial board is kept between the two horn antennas. The distance between the horn antenna and metamaterial is maintained to avoid the near field effects. For reflection measurements, the transmitting and receiving antennas are inclined at a small angle with respect to the normal to the metamaterial surface in order to eliminate the interference of incident and reflected fields. The actual reflection from the metamaterial structure is normalized with respect to a reference metal with the same thickness as the fabricated absorber. In the same way, the transmission measurements are normalized with respect to free space. Different polarizations are produced by rotating the antennas with respect to the Z-axis, and for each polarization, the reflection and transmission data are taken at different incident angles. The fabricated prototype also follows the same trend as simulation results and exhibits bandwidth-enhanced absorption (Fig. 7(c,d)). For TE polarization, A_1_ absorption band exhibits peaks with magnitude greater than 90% from 13.37 GHz to 14.38 GHz (bandwidth of 1.01 GHz) and similarly for A_2_ absorption band extends from 13.30 GHz to 14.20 GHz (bandwidth of 0.9 GHz). However, for both the absorption bands absorption magnitude in the region between both the peaks reduces but maintains the magnitude greater than 80%. For TM polarization, A_1_ exhibits high absorption from 13.4 GHz to 14.37 GHz (bandwidth of 0.97 GHz) and for A_2_, the high absorption band is exhibited from 13.29 GHz to 14.20 GHz (bandwidth of 0.91 GHz). Similar to the TE polarization case, in the middle region of the absorption band, the magnitude reduces but still maintains greater than 80%. The slight difference with the simulation pattern can be attributed to the fabrication tolerances and the difference between the experimental and simulation environments. Further, the angular receptivity of the fabricated absorber is shown for both TE and TM polarizations, and it maintains the bandwidth-enhanced nature with magnitude greater than 80%.

## Conclusion

A metamaterial based bidirectional bandwidth-enhanced absorber is realized numerically and demonstrated experimentally. Metallic patterns for the two sides of the dielectric substrate are identified, and optimized, which can impedance match in both the incident directions and have multiple resonances that combine to produce a resultant bidirectional bandwidth-enhanced absorption. The optimized bidirectional absorber exhibits more than 90% absorption from 13. 40 GHz to 14.25 GHz for the two incident directions. The design evolution elaborates how the proposed structure contributes towards the bidirectional bandwidth-enhanced absorption. The surface current analysis of the absorber is conducted to get an insight of the absorption mechanism. The retrieved effective parameters show the matched impedance as well as elevated loss factor in the structure enabling bidirectional bandwidth-enhanced absorption. Polarization and angular studies show that the structure is polarization insensitive and wide-angle receptive, which maintains bandwidth-enhanced nature as well as high magnitude up to 30°. Finally, all the simulated results are verified experimentally, which shows good agreement between them.

## Supplementary information

Realization of Bidirectional, Bandwidth-Enhanced Metamaterial Absorber for Microwave Applications
