# Supplementary material for: Realization of Bidirectional, Bandwidth-Enhanced Metamaterial Absorber for Microwave Applications
Source: Sci Rep. 2019 Jul 11;9:10058. doi: 10.1038/s41598-019-46464-6 (PMC6624316; doi:10.1038/s41598-019-46464-6)
Supplement: Supplementary file 1 — Realization of Bidirectional, Bandwidth-Enhanced Metamaterial Absorber for Microwave Applications [file 41598_2019_46464_MOESM1_ESM.pdf]

Supplementary Material:

Realization of Bidirectional, Bandwidth-Enhanced Metamaterial Absorber for Microwave Applications

Lincy Stephen[1], Yogesh N.[2], Subramanian V.[1,a)]

[1]Microwave Laboratory, Department of Physics, Indian Institute of Technology Madras, Chennai-600036, India

[2]Department of Nuclear Physics, School of Physical Sciences, University of Madras, Chennai-600025, India

[a)]Corresponding author: E-mail address: manianvs@iitm.ac.in

- **Dimensions of the optimized unit cell**

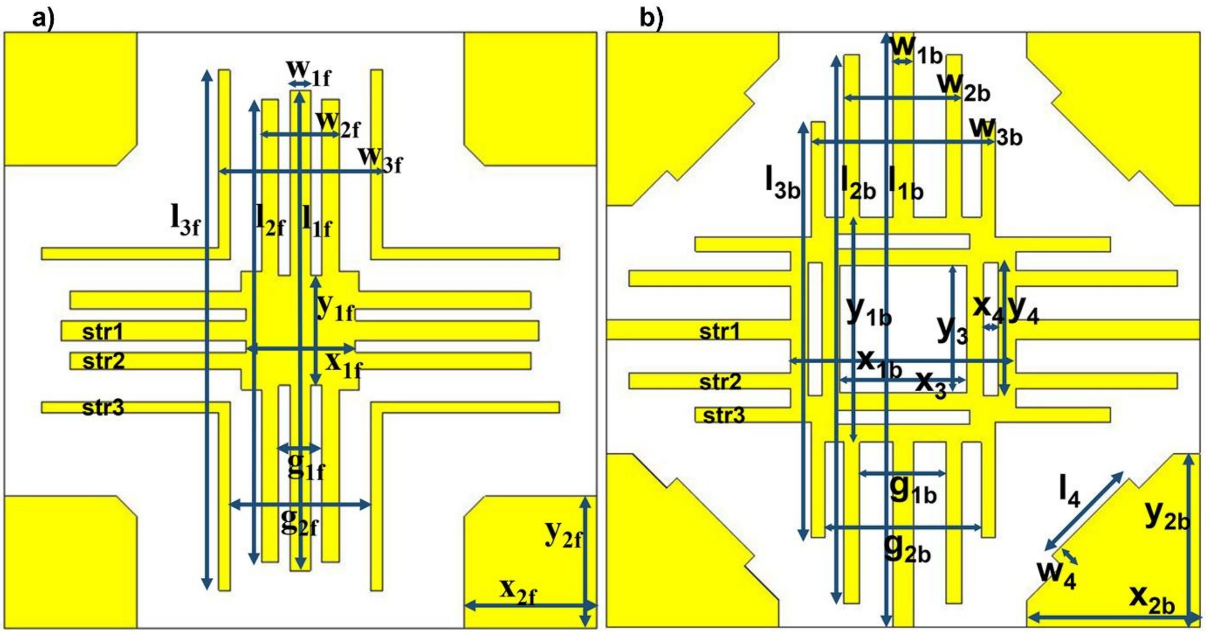

Figure S1. The unit cell of the proposed bidirectional broadband absorber a) front b) back

Front layer:

l_{1f}	w_{1f}	l_{2f}	w_{2f}	l_{3f}	w_{3f}	x_{1f}, y_{1f}	x_{2f}, y_{2f}	g_{1f}	g_{2f}
8.3	0.35	8	1.35	9	2.85	1.9	2.3	0.75	2.45

Back layer:

l_{1b}	w_{1b}	l_{2b}	w_{2b}	l_{3b}	w_{3b}	x_{1b}, y_{1b}	x_{2b}, y_{2b}	g_{1b}	g_{2b}	x_3, y_3	x_4	y_4	l_4	w_4
10.3	0.35	9.5	2.05	7.2	3.2	3.9	3	1.5	2.7	2.2	2.3	0.25	1.9	0.25

All dimensions are in mm.

- **Surface current analysis at absorption frequencies for oblique incidence**

To explore the absorption mechanism at oblique incidence, surface current analysis is carried out at the frequencies where additional absorption peaks are generated.

At 12.56 GHz where a new absorption peak appears in the A_1 spectrum of the TE polarization, the structure exhibits four sets of current flow in the four arms of str1 and str2 in the front and back layers (Fig. S2 (a), (b)). The current flow in the front and back layers are antiparallel to each other in the respective sections resulting in magnetic resonance in the structure. At 11.42 GHz, A_2 spectrum exhibits a peak for TM polarization incidence. At this frequency, there is a current flow existing between the horizontal arms of str1, str2 to the vertical arms of str1, str2 in the front and back layers (Fig. S2 (c), (d)). Similar to the previous frequency, the current flow in the front layer and back layer are antiparallel to each other, resulting in magnetic resonance. Additionally, the str3 in the front and back layers also exhibit similar behaviour contributing to magnetic resonance.

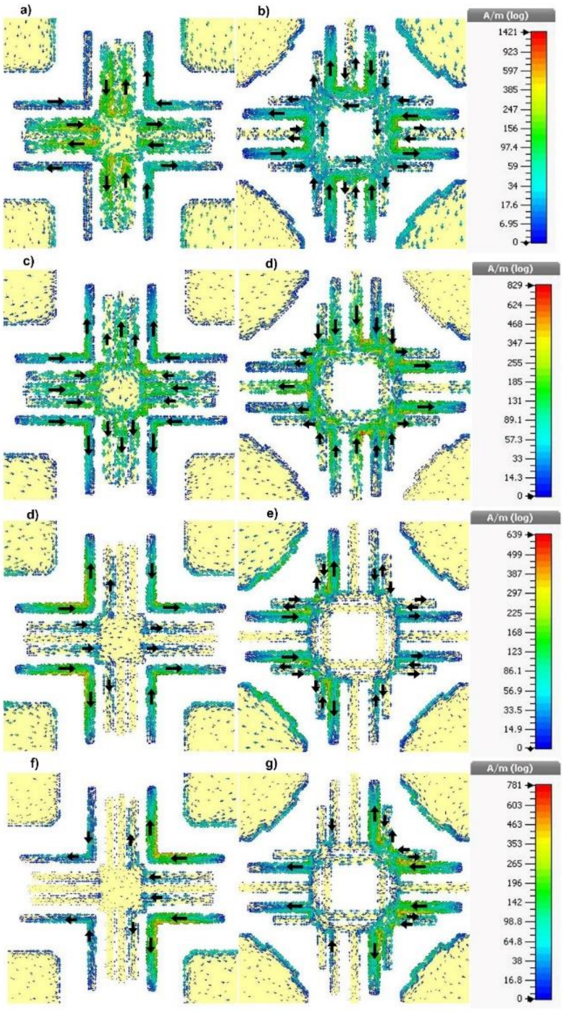

Figure S2. The Surface current distribution, for radiation incident at 30°, at frequencies where additional peaks are observed. At 12.56GHz for A_1 with TE polarization a) front b) back layer,

at 11.42GHz for A_2 with TM polarization c) front layer d) back layer, at 15.77GHz for A_1 with TM polarization e) front layer f) back layer, at 15.77 GHz for A_2 with TM polarization g) front layer h) back layer

At 15.77 GHz, both A_1 and A_2 exhibits peaks for TM polarization incidence. At this frequency, the structures exhibit nonsymmetric current distribution in the layers, i.e. the current density in the one side (with respect to the y-axis) is not equal to that of the other side. In the case of A_1, the sections of str2 and str3 in each quarter of the front and back layers form parallel current distributions resulting in electric resonance (Fig. S2 (e), (f)). Similar to A_1, A_2 also exhibits similar absorption mechanism with parallel currents in str2 and str3 in different sections of the front and back layers (Fig. S2 (g), (h)).
